# CO_2_/N_2_ Separation Properties of Polyimide-Based Mixed-Matrix Membranes Comprising UiO-66 with Various Functionalities

**DOI:** 10.3390/membranes10070154

**Published:** 2020-07-17

**Authors:** Chong Yang Chuah, Junghyun Lee, Juha Song, Tae-Hyun Bae

**Affiliations:** 1 Singapore Membrane Technology Centre, Nanyang Environment and Water Research Institute, Nanyang Technological University, Singapore 637141, Singapore; chongyang.chuah@ntu.edu.sg; 2 School of Chemical and Biomedical Engineering, Nanyang Technological University, Singapore 637459, Singapore; junghyun002@e.ntu.edu.sg (J.L.); songjuha@ntu.edu.sg (J.S.); 3 Department of Chemical and Biomolecular Engineering, Korea Advanced Institute of Science and Technology, Daejeon 34141, Korea

**Keywords:** CO_2_ capture, polyimide, UiO-66, mixed-matrix membrane, pre-synthetic functionalization

## Abstract

Nanocrystalline UiO-66 and its derivatives (containing -NH_2_, -Br, -(OH)_2_) were developed via pre-synthetic functionalization and incorporated into a polyimide membrane to develop a mixed-matrix membrane (MMM) for CO_2_/N_2_ separation. Incorporation of the non-functionalized UiO-66 nanocrystals into the polyimide membrane successfully improved CO_2_ permeability, with a slight decrease in CO_2_/N_2_ selectivity, owing to its large accessible surface area. The addition of other functional groups further improved the CO_2_/N_2_ selectivity of the polymeric membrane, with UiO-66-NH_2_, UiO-66-Br, and UiO-66-(OH)_2_ demonstrating improvements of 12%, 4%, and 17%, respectively. Further evaluation by solubility–diffusivity analysis revealed that the functionalized UiO-66 in MMMs can effectively increase CO_2_ diffusivity while suppressing N_2_ sorption, thus, resulting in improved CO_2_/N_2_ selectivity. Such results imply that the structural tuning of UiO-66 by the incorporation of various functional groups is an effective strategy to improve the CO_2_ separation performance of MMMs.

## 1. Introduction

Carbon capture, storage, and sequestration (CCS) processes have been heavily researched in recent years as a potentially feasible means to minimize the increase in global CO_2_ concentration.

CO_2_ capture accounts for 70% of the total cost of a typical CCS process [1,2,3]. Hence, efficient separation methods are required to remove CO_2_ for mitigating the impact of its emission into the atmosphere. In comparison with conventional gas separation processes, such as cryogenic distillation, absorption, and adsorption, membrane separation processes offer several competitive advantages, such as a reduced plant footprint and greater energy efficient [4,5,6,7,8,9,10,11]. In particular, polymeric membranes are commonly used in such processes due to their well-established synthesis and ease of commercial availability. However, an inevitable trade-off relationship between permeability and selectivity, as evidenced by the so called Robeson upper bound, has proven to be a major drawback of conventional polymeric membranes [12,13]. Meanwhile, zeolites or metal–organic framework (MOF)-based membranes, which demonstrate high gas selectivity, generally show poorer scalability than polymeric membranes due to their inherent brittleness [14,15,16].

The incorporation of porous fillers into polymer films to form mixed-matrix membranes (MMMs) has been explored with the aim of combining the advantages of polymer and porous fillers. In terms of the choice of porous materials, MOFs have attracted substantial research interest due to properties such as their large accessible surface area and micropore volume [17,18,19,20,21]. MOFs are also capable of pre- or post-synthetic functionalization to promote favorable interaction with target gases such as CO_2_, which is highly polarizable [22,23,24]. In addition, the presence of organic moieties in MOFs also ensures better compatibility with the polymer matrices than zeolites, which typically require additional compatibilizers to mitigate the interfacial defects that would otherwise be present between the filler and polymer [25,26,27,28,29].

In this work, we demonstrate the potential utility of nanocrystalline UiO-66 and its derivatives (UiO-66-NH_2_, UiO-66-Br, and UiO-66-(OH)_2_) in a polymeric membrane for CO_2_/N_2_ separation. The presence of zirconium at the metal sites in UiO-66 allows very stable coordination bonding with the ligand, resulting in high stability under humid conditions [30,31,32]. Furthermore, the synthesis of UiO-66 nanocrystals can be conducted using a facile, scalable method [33]. The CO_2_/N_2_ separation performance was further tuned with the incorporation of ligands with various functional groups, namely amine (-NH_2_), bromine (-Br), and hydroxyl (-OH), via pre-synthetic functionalization, as these functional groups interact favorably with CO_2_. In terms of the choice of polymeric membrane, an in-house polyimide, ODPA-TMPDA (the abbreviations will be elaborated in Section 2.1), was used. OPDA-TMPDA ($P_{CO_{2}}=88\;\mathrm{barrer}$) possesses greater CO_2_ permeability than commercial polymers such as Matrimid ($P_{CO_{2}}=10\;\mathrm{barrer}$), polysulfone ($P_{CO_{2}}=5\;\mathrm{barrer}$), and Ultem ($P_{CO_{2}}=2\;\mathrm{barrer}$) [34,35,36,37,38]. Moreover, ODPA-TMPDA can be readily synthesized without a monomer purification process [39,40]. In contrast, the synthesis of 6FDA-based polymers (6FDA = 4,4′-(hexafluoroisopropylidene)diphthalic anhydride) (e.g., 6FDA-DAM (DAM = 2,4,6-trimethyl-*m*-phenylenediamine), $P_{CO_{2}}=681\;\mathrm{barrer}$) and PIM-1 (polymer of intrinsic microporosity-1) ($P_{CO_{2}}=5120\;\mathrm{barrer}$) typically requires the monomers to be purified before polymer synthesis to allow the production of those polymers with a high average molecular weight in order to develop membrane with high intrinsic CO_2_ permeability [41,42,43,44]. It should be noted that the CO_2_ permeabilities indicated above are obtained at the condition that is reasonably close to the measurement condition used in this work (35 °C and 1 bar feed pressure, as stated in Section 2.4.3.). Finally, the effect of different functional groups in UiO-66 on CO_2_/N_2_ separation performance was systematically studied.

## 2. Materials and Methods

### 2.1. Materials

2-Bromoterephthalic acid, 2-aminoterephthalic acid, 2-5-dihydroxyterephthalic acid, 2,4,6-trimethyl-*m*-phenylenediamine (TMPDA), 4,4′-oxydiphthalic anhydride (ODPA), acetic anhydride (Ac_2_O), terephthalic acid, triethylamine (TEA), and zirconium(IV) chloride (ZrCl_4_) were purchased from Sigma Aldrich (Singapore). Chloroform, dimethylformamide (DMF), methanol, *N*,*N*-dimethylacetamide (DMAc), and hydrochloric acid (HCl, 37%) were purchased from VWR (Singapore). All other chemicals were used as received without further purification.

### 2.2. Synthesis of MOFs (UiO-66 and Its Derivatives) and Polymer (ODPA-TMPDA)

**Figure 1:**
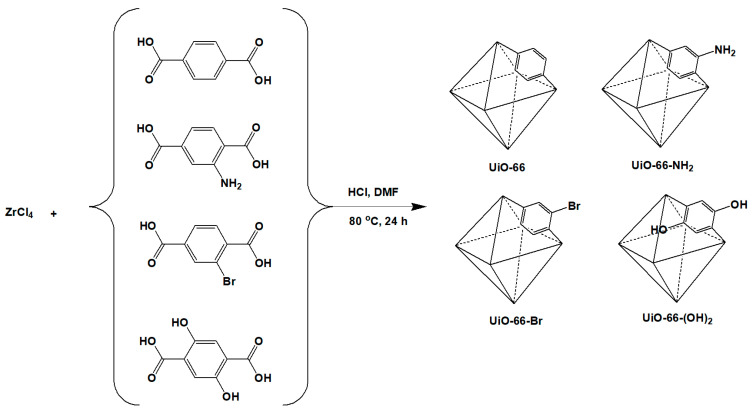
Synthesis scheme of nanocrystalline UiO-66 and its derivatives.

**Figure 2:**
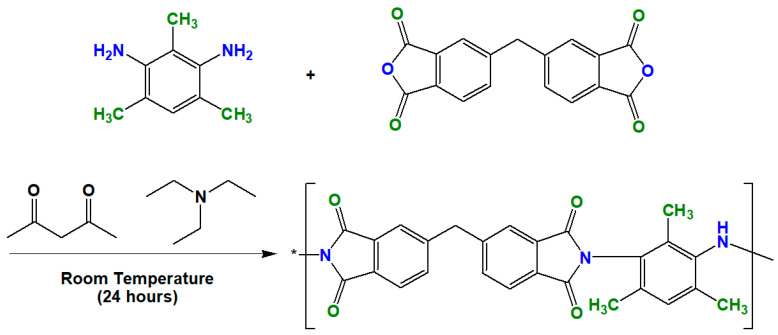
Synthesis of ODPA-TMPDA.

UiO-66 nanocrystals were synthesized by the experimental procedure described as follows, with some modifications [33]. In a round-bottom flask, 1.25 g of ZrCl_4_, 50 mL of DMF and 10 mL of HCl were loaded, and the resulting mixture was sonicated for at least 20 min to ensure that all reactants were completely dissolved and mixed. In a separate flask, 1.23 g of terephthalic acid and 100 mL of DMF were loaded. The contents of this flask were then poured into the above mixture, followed by an additional sonication for at least 20 min. This was followed by heating at 80 °C for 24 h to ensure an effective formation of UiO-66 particles. The precipitated particles were washed and centrifuged with DMF and methanol to remove the unreacted impurities. Before characterization, the samples were dried in a vacuum at 60 °C overnight. For the synthesis of UiO-66-NH_2_, UiO-66-Br, and UiO-66-(OH)_2_, the porous materials were prepared by a similar protocol to that described above, and 1.34 g of 2-aminoterephthalic acid, 1.84 g of 2-bromoterephthalic acid, and 1.47 g of 2.5-dihydroxyterephthalic acid were added to the solution. The reaction scheme was summarized in Figure 1. The synthesis of ODPA-TMPDA polymer, on the other hand, is conducted based on the procedure as described elsewhere [45]. The reaction scheme is provided in Figure 2.

### 2.3. Membrane Fabrication

The membranes were fabricated via a solution-casting method to form a dense membrane film. First, UiO-66 and its derivatives were dispersed in chloroform. The dispersion of porous fillers in the suspension was improved with a sonication horn before the polymer was added into the solution. This step is required for the aggregated nanocrystals of UiO-66 and its derivatives to be dispersed readily in chloroform. To minimize solvent evaporation of chloroform during the sonication process (due to its low boiling point), an ice bath was used. The resulting dope solution was stirred for at least 24 h. Next, membranes were formed by casting on a glass plate, and the thickness was controlled using a casting knife, such that the resulting membrane thickness can be in the range of 50−70 μm. The casting was conducted in a glove bag in an environment filled with chloroform vapor to minimize rapid solvent evaporation, after which the membranes were left undisturbed for 4 to 5 h. The membranes were then annealed at 120 °C in a vacuum oven after ensuring that they had peeled off from the glass plate.

### 2.4. Characterization

#### 2.4.1. Characterization of Nanocrystalline UiO-66 and Its Derivative

A volumetric gas sorption analyzer (iSorbHP1, Quantachrome, Boynton Beach, FL, USA) was used to determine CO_2_ and N_2_ adsorption on nanocrystalline UiO-66 and its derivatives. The samples were activated at 120 °C for 1 day to remove the residual solvents. Measurements of gas adsorption isotherms at 25 and 35 °C were conducted at pressures from 0 to 1 bar, during which a water circulator was used to ensure that the measurement environment remained in isothermal conditions. Considering the shape of the adsorption curve, the single-site Langmuir equation (Equation (1)) was deemed appropriate to fit the isotherm with a sufficiently high R^2^ value [46,47].

(1) $$q=\frac{q_{sat}bp}{1+bp}$$

In Equation (1), *q*, *q_sat_*, *b*, and *p* are the adsorption quantity (mmol/g), saturation loading (mmol/g), Langmuir constant (bar^−1^), and pressure (bar), respectively. The CO_2_/N_2_ selectivity of UiO-66 and its derivative can be calculated using the ideal adsorbed solution theory (IAST) [48], expressed by Equation (2):

(2) $$\mathrm{Selectivity}=\frac{x_{1}/x_{2}}{y_{1}/y_{2}}$$

where *x*_1_, *x*_2_—the mole fraction of the adsorbed phase and *y*_1_, *y*_2_—the mole fraction of the gas phase. The isosteric heat of adsorption, −*Q*_st_ for CO_2_ and N_2_, was evaluated using the Clausius–Clapeyron equation (Equation 3), with *p*, *T*, and *q* denoting pressure (bar), absolute temperature (K), and amount adsorbed (mmol/g), respectively. An explicit analytical solution for the calculation of −*Q_st_* that uses a single-site Langmuir equation has been derived, and it has been observed that −*Q_st_* is a weak function of temperature.

(3) $$-Q_{st}=RT^{2}{\left(\frac{\partial \ln P}{\partial T}\right)}_{q}$$

N_2_ physisorption isotherm: The porosities of UiO-66 and its derivatives were measured via N_2_ physisorption analysis (77 K), under the conditions specified above, using a volumetric gas sorption analyzer (NOVATouch LX2, Quantachrome, Boynton Beach, FL, USA).

Powdered X-ray diffraction (PXRD; Advanced D8, Bruker, Billerica, MA, USA): PXRD was used to verify the crystallinity of the powdered samples. The samples were measured at ambient conditions, with 2*θ* scanned from 5° to 40° (step size of 0.02°) using a Cu-Kα radiation (1.5148 Å) diffractor.

Field-emission scanning electron microscopy (FESEM; JSM6701, JEOL, Akishima, Tokyo, Japan): FESEM was performed to examine the structural morphology of nanocrystalline UiO-66 and its derivatives. The accelerating voltage was set at 5 kV.

Fourier-transformed infrared (FTIR) spectroscopy: FTIR was conducted in the range of 4000−450 cm^−1^ (resolution of 4 cm^−1^) to identify the functional groups of the porous materials (IRPrestige-21, Shimadzu, Kyoto, Japan).

Elemental analysis (Vario EL III CHNS Elemental Analyzer, Elementar, Langenselbold, Germany): Elemental analysis was used to determine the elemental composition of nanocrystalline UiO-66 and its derivatives. The Br content in UiO-66-Br was estimated via FESEM equipped with energy-dispersive X-ray (EDX) spectroscopy.

Thermal stability: The thermal stabilities of the nanocrystalline UiO-66 and its derivatives were measured via thermogravimetric analysis (SDT Q600 TGA, TA Instrument, New Castle, DE, USA), under a temperature scan from 40 to 800 °C at the ramping rate of 10 °C/min. Prior to the analysis, the samples are purged at 120 °C under pure nitrogen flow (flow rate set at 100 mL/min) for 8 h to minimize the effect of the residual solvents on the TGA curve.

#### 2.4.2. Characterization of Mixed-Matrix Membranes

FESEM was used to investigate the cross-sectional morphologies of membranes under uniform accelerating voltage conditions. The membranes were fractured before Platinum coating (with the use of liquid nitrogen) to preserve the overall morphologies. FTIR and XRD analyses were conducted with the same settings as above to investigate the properties of the membranes. Similarly, thermogravimetric analysis was used to investigate the thermal stability of the membranes with the same settings as mentioned above. An analytical balance (ME204, Mettler Toledo, Columbus, OH, USA) equipped with a density kit was used to determine the membranes’ densities. This measurement was conducted by computing the difference in the mass of the samples in an auxiliary liquid (ethanol) and air via Archimedes’ principle. The density of the membrane, *ρ*, can be computed from Equation (4):

(4) $$\rho =\frac{A}{A-B}\left(\rho_{L}-\rho_{A}\right)+\rho_{A}$$

In this equation, A—mass of the membrane sample in air; B—mass of the membrane sample in the auxiliary liquid; $\rho_{L}$—density of the auxiliary liquid; $\rho_{A}$—density of air. The calculated value can be accurately determined up to 4 decimal places, based on the precision of the analytical balance.

#### 2.4.3. Gas Permeation Test

A gas permeation test (GTR-11, GTR Tec Corporation, Kyoto, Japan) was carried out using a constant pressure-variable volume system. The gases (CO_2_/N_2_ test gas: 80 vol% N_2_ (99.9995%) and 20 vol% CO_2_ (99.8%) and helium (99.9995%)) were purchased from Airliquide Singapore Pte. Ltd. The membranes were mounted onto the gas permeation cell with the aid of vacuum grease. To minimize the potential contamination of the vacuum grease onto the desired permeation area (1.77 cm^2^) in the permeation cell, the membrane that is mounted onto the permeation cell is prepared in such a way that the membrane area is larger than the permeation area. Throughout the measurement, a uniform temperature of 35 °C was maintained with a temperature controller. Using a mass flow controller, the test gas and helium were continuously supplied upstream and downstream of the membrane, respectively. At set time intervals, the downstream permeated gas was swept by helium and sent to the gas chromatograph to calculate the composition of the gas stream. This process continued until it was ensured that the permeated gas’ concentration did not fluctuate substantially over a designated time period. The gas permeability and selectivity were calculated based on the concentration of the permeated gas. The equation of permeability, *P*, can be written as shown below (5). The parameters *q*, *l*, *a*, *p*, and *t* are the concentration (of CO_2_ and N_2_ gas calculated from gas chromatography), the membrane thickness, the permeation area, the pressure, and the time, respectively. To ensure sufficient reproducibility of the result, at least three different samples were measured.

(5) $$P=\frac{ql}{apt}$$

#### 2.4.4. Gas Adsorption Analysis

The adsorption properties of CO_2_ and N_2_ gases in each membrane were measured under identical conditions using a volumetric gas sorption analyzer (iSorbHP1, Quantachrome, Boynton Beach, FL, USA). All membranes were outgassed at 120 °C for 1 day. The CO_2_ and N_2_ adsorption isotherms were fitted using the single-site Langmuir equation as elaborated in Equation (1). This was followed by the calculation of the solubility, *S*, of CO_2_ and N_2_ in the respective membranes. In Equation (6), *q*, *ρ*, and *p* are defined as the gas adsorbed per unit membrane mass, the membrane’s density, and the specified pressure, respectively. The diffusivity, *D*, of gas in the membrane can be calculated using the relationship between permeability and solubility because the solution–diffusion mechanism is the transport mechanism in a dense membrane.

(6) $$S=\frac{q\rho }{p}$$

#### 2.4.5. Filler Enhancement Index

Equation (7), which evaluates the parameter of filler enhancement index (*F_index_*), was used to calculate the performance of the MMMs. In this expression, *P_filled_* and *P_unfilled_* are defined as the permeability of MMM and pure polymeric membrane; *α_filled_* and *α_unfilled_* are the CO_2_/N_2_ selectivity of MMM and pure polymeric membrane; *η* is the enhancement coefficient, which is defined as 2.888 based on the slope of the Robeson upper bound published in 2008. This parameter was defined for CO_2_/N_2_ in a 2008 study to assist in quantifying the effectiveness of fillers in MMMs [4].

(7) $$F_{index}=\ln\left(\frac{P_{filled}}{P_{unfilled}}\right)+\eta \ln\left(\frac{\alpha_{filled}}{\alpha_{unfilled}}\right)$$

## 3. Results and Discussion

### 3.1. Synthesis of Nanocrystalline UiO-66 and Its Derivatives

**Figure 3:**
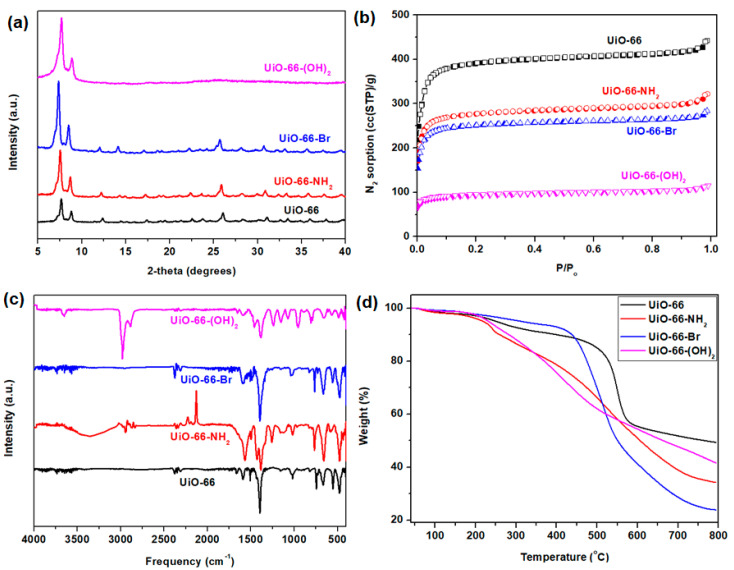
(**a**) Powdered X-ray diffraction (PXRD) pattern; (**b**) N_2_ adsorption–desorption isotherms at 77 K (open and closed symbols describe the adsorption and desorption isotherms); (**c**) FTIR curves; (**d**) thermogravimetric curves of UiO-66 and its derivatives.

**Figure 4:**
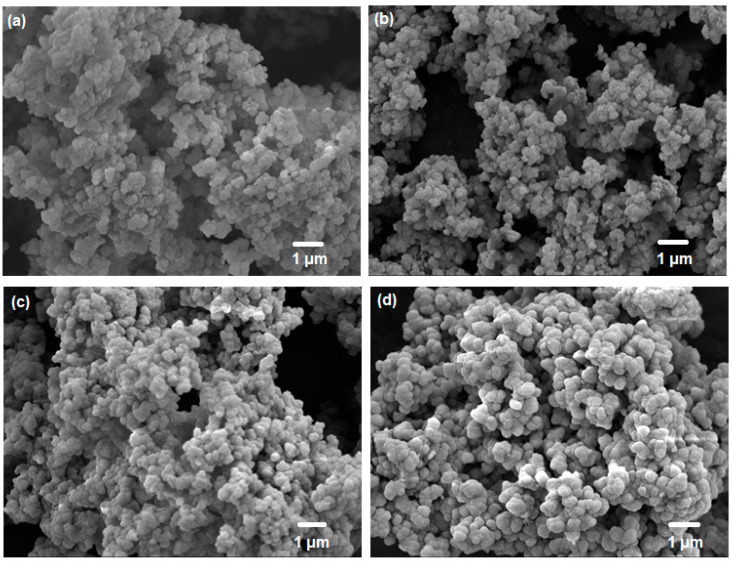
FESEM images of (**a**) UiO-66; (**b**) UiO-66-NH_2_; (**c**) UiO-66-Br; (**d**) UiO-66-(OH)_2_.

First, the PXRD patterns were inspected to verify the crystallinity of the UiO-66 nanocrystals (Figure 3a). A comparison of UiO-66 and its derivatives shows that the overall structural crystallinity remained intact, despite the use of different ligands. The peaks observed here are consistent with the diffraction peaks previously reported for UiO-66 [49,50,51,52,53]. Next, the N_2_ adsorption–desorption isotherm measured at 77 K (Figure 3b) clearly depicted that the UiO-66 nanocrystals possess high N_2_ sorption at low P/P_o_. This implies that these nanocrystals possess large micropore volumes, as summarized in Table 1. However, it can be expected that after the incorporation of ligands with different functional groups (via pre-synthetic functionalization), the overall porosity of the framework would decrease substantially. Indeed, the N_2_ adsorption–desorption measurements confirm the decreases in overall porosity with the various functional groups. As expected, the introduction of difunctional groups causes a sharper decrease in the accessible surface area than modification with monofunctional groups. Comparing amine (-NH_2_) and bromine (-Br) functional groups, UiO-66-Br suffers a slight decrease in accessible surface area compared with UiO-66-NH_2_, consistent with the larger atomic size of -Br. Further verification of the presence of -NH_2_ and -Br functional groups was conducted via elemental analysis and EDX (Figure S1), and the results are summarized in Table S1 (the theoretical values of the elemental composition are supplemented in Table S2). FTIR analysis of UiO-66 and its derivatives demonstrated that the presence of different functional groups did not influence the FTIR spectrum (Figure 3c). The spectrum of UiO-66 is similar to that reported in the literature [54]. A clearly observable O-H stretch as well as N-H stretch at around 3000 and 3300 cm^−1^, respectively, for UiO-66-(OH)_2_ and UiO-66-NH_2_ indicated the unmistakable presence of 2,5-dihydroxyterephthalate and 2-aminoterephthalate in each sample. As for the thermal stability of UiO-66 and its derivatives, thermogravimetric analysis (Figure 3d) demonstrated that the overall structure of UiO-66 and UiO-66-Br remained thermally stable up to 550 and 500 °C, respectively, whereas UiO-66-NH_2_ and UiO-66-(OH)_2_ showed a substantial weight loss after 200 °C. Thus, for the subsequent experiments (gas adsorption and membrane annealing), the activation condition of all nanoporous materials were set at 120 °C. In addition, it should be noted that the nanoporous materials that are used in MMM for gas separation should have sufficiently small particle sizes [55]. Thus, the morphologies of UiO-66 and its derivatives were verified via FESEM (Figure 4). Based on the images, the particle sizes generally ranged from 300 to 500 nm, which is sufficiently small for these particles to be used in gas separation.

### 3.2. CO_2_ and N_2_ Adsorption by UiO-66 and Its Derivatives

**Figure 5:**
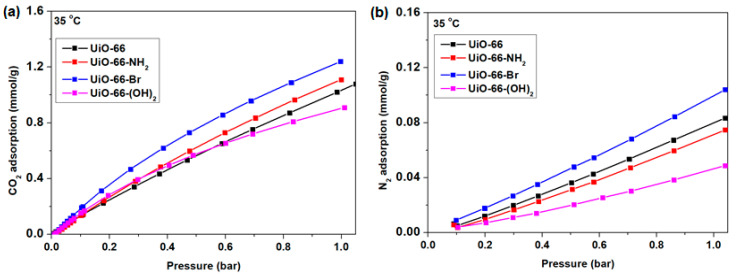
(**a**) CO_2_ and (**b**) N_2_ adsorption by nanocrystalline UiO-66 nanocrystals and its derivatives at 35 °C.

The properties of UiO-66 and its derivatives were further characterized by the measurement of CO_2_ and N_2_ adsorption at 35 °C, with the results summarized in Figure 5. The CO_2_ and N_2_ adsorption isotherms at 25 °C were also acquired and are summarized in Figure S2a,b. The fitting parameters for CO_2_ and N_2_ adsorption are summarized in Table S3 (25 °C) and Table S4 (35 °C) respectively. In general, UiO-66 and all of its derivatives proved to be able to preferentially adsorb CO_2_ relative to N_2_, consistent with the fact that the former gas possesses a higher quadrupole moment (4.3 × 10^−26^ esu cm^2^ vs. 1.5 × 10^−26^ esu cm^2^) and polarizability (29.11 × 10^−25^ cm^3^ vs. 17.4 × 10^−25^ cm^3^) [56]. The presence of zirconium at the metal sites and the functional groups in the ligands (-NH_2_, -Br, -(OH)_2_) both promoted favorable interaction with CO_2_. Notably, UiO-66-NH_2_ and UiO-66-Br showed a clear enhancement of CO_2_ adsorption capability relative to UiO-66, even though they possessed a smaller surface area. The adsorption capability was poorly correlated with the accessible surface areas, possibly because none of the samples had reached CO_2_ adsorption saturation at 1 bar. In contrast, although UiO-66-(OH)_2_ showed reasonably high CO_2_ adsorption at low partial pressure, due to its low surface area (318 m^2^/g) relative to the other samples, the slope of the isotherm indicated a faster equilibrium saturation of CO_2_ adsorption than for the other adsorbents [51,57]. Nevertheless, in terms of the applicability for CO_2_ adsorption in the field of post-combustion CO_2_ capture, which is conducted at low partial pressure of CO_2_ [58], UiO-66-NH_2_, UiO-66-Br, and UiO-66-(OH)_2_ each demonstrated better CO_2_ adsorption performance than UiO-66. Each of these observations are supported by the higher isosteric heat of adsorption of CO_2_ on the functionalized adsorbents (Figure S2c) together with higher IAST CO_2_/N_2_ selectivity (Figure S2d), which indicates that the use of ligands with any of the various functional groups in this work is a feasible strategy to improve the CO_2_ adsorption performance compared with UiO-66.

### 3.3. Fabrication of Mixed-matrix Membranes

**Figure 6:**
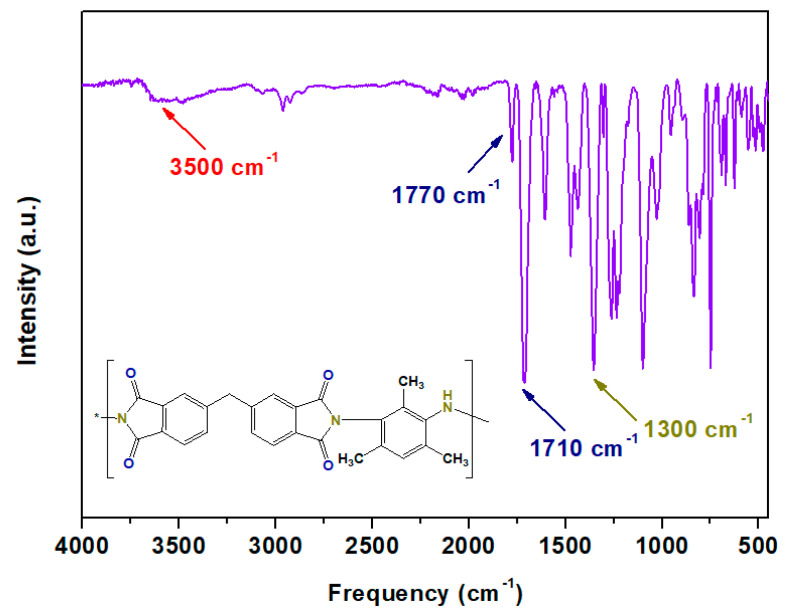
FTIR spectrum of ODPA-TMPDA polymer.

In this study, in-house-made polyimide (ODPA-TMPDA) was used as the polymeric membrane for the gas separation process. Successful synthesis of ODPA-TMPDA was verified from its FTIR spectrum (Figure 6), which exhibits the characteristic asymmetric and symmetric stretching (1770 and 1710 cm^−1^) of C=O as well as the stretching of C-N (1300 cm^−1^). The corresponding colors of the functional groups are also indicated in the figure for guidance. The most important evidence of successful formation of ODPA-TMPDA is the band at 3500 cm^−1^ (i.e., O-H absorption band), corresponding to residual unreacted polyamic acid. As described in the section on synthesis procedure [45], the ODPA-TMPDA polymer was formed in two steps: (1) formation of polyamic acid via condensation reaction and (2) chemical imidization of polyamic acid. This band was not detected in this work, implying the imidization was completely done. The overall features of the spectrum are comparable to those of previously reported spectra [39,40,59].

**Figure 7:**
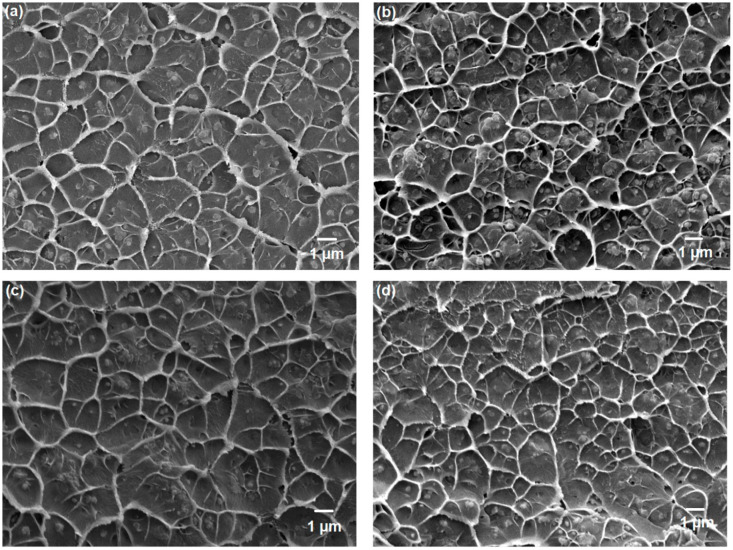

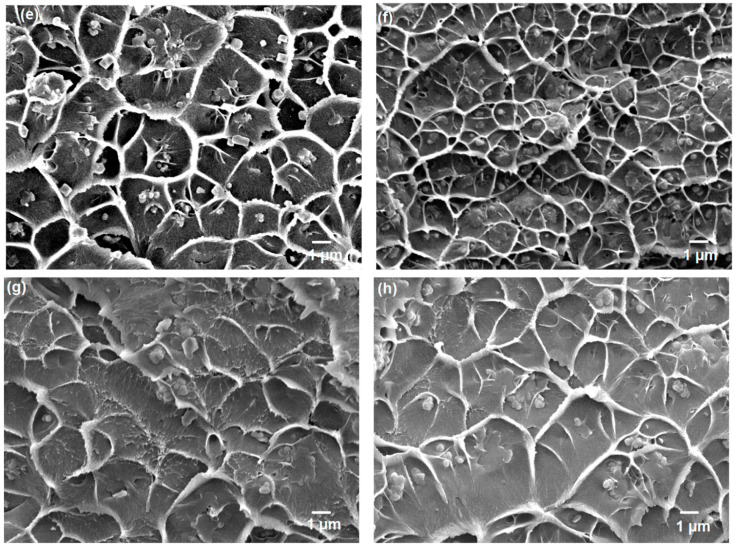
FESEM images of (**a**) 10 wt% UiO-66; (**b**) 20 wt% UiO-66; (**c**) 10 wt% UiO-66-NH_2_; (**d**) 20 wt% UiO-66-NH_2_; (**e**) 10 wt% UiO-66-Br; (**f**) 20 wt% UiO-66-Br; (**g**) 10 wt% UiO-66-(OH)_2_; (**h**) 20 wt% UiO-66-(OH)_2_ in ODPA-TMPDA membrane.

Hence, MMMs containing UiO-66, UiO-66-NH_2_, UiO-66-Br, and UiO-66-(OH)_2_ with 10 and 20 wt% loading were developed in this work. The FTIR spectra of the MMMs indicate that the structural properties of the polyimide membrane remained intact (Figure S3). In addition, the XRD patterns of the MMMs indicate that the crystallinity of UiO-66 and its derivatives remained unaffected after annealing (Figure S4). Hence, the cross-sectional morphologies were further investigated by FESEM (Figure 7). The sieve-in-a-cage morphology, which is common in zeolite/polymer MMMs, was not observed in this work [60]. The presence of organic ligands in UiO-66, UiO-66-NH_2_, UiO-66-Br, and UiO-66-(OH)_2_ improves the polymer/filler compatibility. The use of small particles in this study was also advantageous because it created large interfacial areas between the filler and polymer [41,61]. Thermogravimetric analysis of the pure polymer and MMMs verified that the incorporation of nanoporous materials did not affect the thermal stability of the polymer (Figure S5). The initial drop in weight loss is attributed to the removal of residual solvent present in the sample. For the MMMs, because the thermal stability of the filler is weaker than that of the polymer matrices, a two-stage weight loss was observed. This observation is consistent with a previous study [22].

### 3.4. Gas Permeation Properties

The gas permeation properties of the membranes were measured at the upstream pressure of 1 bar of CO_2_ (20/80 mixture) at 35 °C (Table 2). The presence of 20 wt% UiO-66 in the polymeric membrane was found to improve the overall CO_2_ permeability by 92%, but with a marginal decrease in CO_2_/N_2_ selectivity. The large micropore volume of UiO-66 presumably allowed rapid transport of both CO_2_ and N_2_ molecules through the MMM with minimal resistance. However, due to the relatively poor CO_2_/N_2_ selectivity of UiO-66, it was anticipated that the incorporation of the above-tested derivatives of UiO-66 in MMMs would give better gas separation performance. Indeed, the gas permeation results confirm that UiO-66-NH_2_, UiO-66-Br, and UiO-66-(OH)_2_ improved the CO_2_/N_2_ selectivity by 12%, 4%, and 17%, respectively, which is consistent with the IAST calculation (Figure S1d), which predicted that UiO-66-(OH)_2_ would demonstrate the highest improvement in selectivity among the UiO-66 derivatives. Nevertheless, considering the overall performance in terms of both CO_2_ permeability and CO_2_/N_2_ selectivity, 20 wt% UiO-66-Br demonstrates the highest CO_2_ permeability along with attractive CO_2_/N_2_ selectivity.

**Figure 8:**
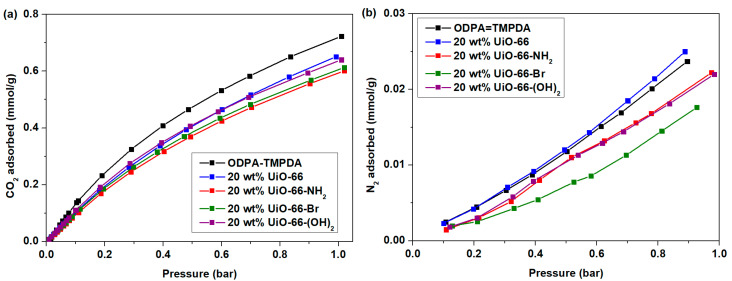
(**a**) CO_2_ and (**b**) N_2_ isotherm of ODPA-TMPDA membrane and mixed-matrix membrane containing UiO-66, UiO-66-NH_2_, UiO-66-Br and UiO-66-(OH)_2_ at 20 wt% loading.

The solubility–diffusivity of CO_2_ and N_2_ in MMMs was then quantitatively analyzed. The solubility of gas in the membranes was determined by measuring the pure-component CO_2_ and N_2_ adsorption isotherms at 35 °C, with the data summarized in Figure 8. The fitting parameters for CO_2_ and N_2_ adsorption by the membranes are compiled in Table S5. Table 3 summarizes the quantitative solubility–diffusivity data for CO_2_ and N_2_ in the membranes. Based on the gas adsorption data (which corresponds to the analysis of solubility), at the point of interest (CO_2_ at 0.2 bar), the adsorption performance is slightly inferior to the pristine membrane without nanocrystals. Nevertheless, at 0.8 bar of N_2_ (point of interest in this study), the incorporation of UiO-66 and its derivatives substantially suppressed the N_2_ solubility, which is consistent with the behavior of other porous materials in membranes, as reported in previous studies [22,40,59]. In contrast, it was found that the diffusivities of both CO_2_ and N_2_ dramatically increased upon incorporation of nanocrystalline UiO-66 and its derivatives, which possess large pore volumes, resulting in improved gas permeabilities of MMMs.

Besides, the CO_2_/N_2_ separation properties of our membranes are also compared with the literature data, where UiO-66 and its derivative are used as the fillers in the fabrication of MMM. In general, as summarized in the gas permeation data (Table 2) and Table 4, UiO-66 is incapable of improving the CO_2_/N_2_ selectivity to a substantial extent, unless rubbery polymer (PEBA) is used, as reported by Shen et al. [51]. This is plausibly attributed to the high chain mobility of the rubbery polymer, which minimize the formation of interfacial nanogaps more effectively than the glassy polymers [61]. Nevertheless, although the performance of UiO-66 in PEBA looks promising, it is noteworthy that membrane should be fabricated on a porous support owing to its poor mechanical stability [62,63,64], thus, limiting potential utility in large scale membrane production. In order to ensure a clear enhancement in CO_2_/N_2_ selectivity of glassy polymer membrane, functionalization of UiO-66 to allow favorable CO_2_ adsorption is deemed necessary.

With reference to the CO_2_/N_2_ separation performance reported in Table 4, an empirical metric (*F*_index_) was used to compute the effectiveness of the fillers in MMM, considering the fact that different polymers were used as the matrices in membranes. In this work, the most promising performance was that of UiO-66-Br at 20 wt% loading, with *F_index_* = 0.94, a performance close to the Robeson upper bound for CO_2_/N_2_ separation [12], as plotted in Figure S6. In contrast, the value of *F_index_* for UiO-66 was only 0.09 at 10 wt% and 0.55 at 20 wt% loading, lower than for any other fillers investigated in this work. Notably, the *F*_index_ of 0.94 for the case of UiO-66-Br is found to be higher than most of the reported literature data, with the exception of the incorporation of azo-UiO-66 at 10 wt% loading in Matrimid membrane [65] and UiO-66-H (20 wt%) and UiO-66-NH_2_ (10 wt%) in PIM-1 membrane [66]. However, it should be noted that the data in the above studies were obtained from the pure gas permeation test, which does not reflect the practical conditions. For example, the partial pressure of CO_2_ in the feed is considerably low (<20 vol%) in a typical post-combustion CO_2_ capture process [58,67], as compared to volume fraction of 50 vol% (of CO_2_) used in several studies [51,66]. Thus, evidently, the measurement of UiO-66-NH_2_ (10 wt%) in PIM-1 membrane under the mixed-gas condition has led to a clear 54% decrease in *F*_index_ from 1.48 to 0.67, as shown in Table 4. Hence, it is generally important to conduct the measurement under the mixed-gas condition rather than pure gas testing in order to demonstrate a clear illustration of the gas separation performance of porous materials in MMM.

## 4. Conclusions

Nanocrystalline UiO66 and its derivatives were developed via pre-synthetic functionalization with ZrCl_4_, and these porous fillers were used to develop MMMs for the analysis of CO_2_/N_2_ separation. It was observed that the addition of UiO-66 nanocrystals successfully improved CO_2_ permeability but with a slight dip in CO_2_/N_2_ selectivity. Thus, derivatives of UiO-66 with different functional groups (-NH_2_, -Br, and -(OH)_2_) were prepared and evaluated under similar conditions. All of these UiO-66 derivatives were found to improve the CO_2_/N_2_ selectivity, which was consistent with the calculated IAST of these porous materials together with the experimental results of a gas permeation test, where the particles were incorporated into an MMM. Further solubility–diffusivity analysis indicated that the addition of UiO-66 derivatives with different functional groups suppressed the solubility of N_2_, which led, in turn, to enhanced CO_2_/N_2_ selectivity. Based on our findings, UiO-66-Br at 20 wt% loading is capable of improving both the CO_2_ permeability and CO_2_/N_2_ selectivity of MMMs for gas separation, with performance close to the reported upper bound. Future efforts can be devoted to realizing a practically attractive performance by employing high-performance polymer matrices (e.g., 6FDA-based polyimides and PIM-1) in MMM fabrication.

## Figures and Tables

**Table 1:** Surface areas and pore volumes of UiO-66 and its derivatives, determined by N_2_ physisorption at 77 K.

Sample	S_BET_^(a)^ (m^2^/g)	S_LANG_^(a)^ (m^2^/g)	S_micro_^(b)^ (m^2^/g)	V_micro_^(b)^ (cc/g)	V_total_^(c)^ (cc/g)
UiO-66	1733	2266	1662	0.809	0.938
UiO-66-NH_2_	1218	1599	1160	0.569	0.682
UiO-66-Br	851	1117	818	0.380	0.440
UiO-66-(OH)_2_	318	418	293	0.136	0.178

^(a)^ BET surface area and Langmuir surface area (S_BET_ and S_LANG_) were determined at P/P_o_ = 0.05–0.2; ^(b)^ Micropore surface area and micropore volume (S_micro_ and V_micro_) were determined at P/P_o_ = 0.4–0.6 using *t*-plot method; ^(c)^ Total pore volume (V_total_) was determined at P/P_o_ = 0.99.

**Table 2:** Permeation results of the membranes at 35 °C at the feed pressure of 1 bar (or 10^5^ Pa) CO_2_/N_2_ mixture (20 vol%/80 vol%) ^(a)^.

Membrane	CO_2_ Permeability (Barrer) ^(b)^	CO_2_/N_2_ Selectivity
ODPA-TMPDA	88 ± 2	33.1 ± 1.2
10 wt% UiO-66	142 ± 5	29.0 ± 0.4
20 wt% UiO-66	169 ± 2	31.9 ± 0.2
10 wt% UiO-66-NH_2_	129 ± 3	36.1 ± 0.8
20 wt% UiO-66-NH_2_	142 ± 1	37.1 ± 2.3
10 wt% UiO-66-Br	158 ± 2	33.7 ± 1.0
20 wt% UiO-66-Br	200 ± 4	34.5 ± 1.9
10 wt% UiO-66-(OH)_2_	98 ± 2	35.2 ± 0.8
20 wt% UiO-66-(OH)_2_	125 ± 4	38.9 ± 0.9

^(a)^ The overall thickness of the membrane is determined to be ranging from 50 to 70 μm, based on the measurement from a micrometer screw gauge. ^(b)^ 1 Barrer = 3.35 × 10 ^−16^ mol-m/m^2^-s-Pa.

**Table 3:** Solubility–diffusivity data of CO_2_ and N_2_ on membranes measured at 35 °C and total feed pressure of 1 bar. The pressure point is determined to be 0.2 bar for CO_2_ and 0.8 bar for N_2._

Membrane	Density (g/cm^3^)	CO_2_ Solubility (mol/m^3^.bar)	CO_2_ Diffusivity, × 10^−12^ (m^2^/s)	N_2_ Solubility (mol/m^3^.bar)	N_2_ Diffusivity, × 10^−12^ (m^2^/s)
ODPA-TMPDA	1.286	1526	1.96	32.1	2.81
20 wt% UiO-66	1.210	1164	4.92	31.5	5.71
20 wt% UiO-66-NH_2_	1.330	1172	4.11	28.1	4.62
20 wt% UiO-66-Br	1.354	1256	5.40	22.5	8.72
20 wt% UiO-66-(OH)_2_	1.380	1385	3.06	29.3	3.72

**Table 4:** Summary of gas permeation results of the mixed-matrix membranes that utilize UiO-66 and its derivatives ^(a)^.

Filler	Polymer	Filler Loading (wt%) ^(b)^	Separation Performance						*F*_index_	Yr (Ref.)
			Testing Condition		*P*(CO_2_) Barrer	% En.	α (CO_2_/N_2_)	% En.		
			Pressure (bar)	Temp. (°C)						
**UiO-66**	PEBA	10	- ^(c)^	25	96.3	87.0	56.6	34.4	1.48	16′ [51]
**UiO-66**	PEBA	10	- ^(c, d)^	25	139.7	171	61.1	45.1	2.07	16′ [51]
**UiO-66**	PSF	20	3	35	16	186	26.2	−11.0	0.71	16′ [68]
**UiO-66-NH_2_**	PEBA	20	- ^(c)^	25	87.0	68.9	66.1	57.0	1.82	16′ [51]
**UiO-66-NH_2_**	PEBA	20	- ^(c, d)^	25	130.2	153	72.2	71.5	2.48	16′ [51]
**UiO-66-ref**	PIM-1	20	4	25	6981	128.6	13.0	−19.3	0.21	17′ [66]
**UiO-66**	PIM-1	23.1	1	25	7610	59.5	20.7	−5.1	0.31	17′ [50]
**UiO-66**	PIM-1 (MeOH treated)	23.1	1	25	9980	109.2	21.6	−0.9	0.71	17′ [50]
**UiO-66-COOH**	PIM-1	23.1	1	25	5300	11.1	20	−8.26	−0.14	17′ [50]
**UiO-66-H**	PIM-1	20	4	25	2606	−14.7	24.6	52.8	1.07	17′ [66]
**UiO-66-NH_2_**	PIM-1	9.1	1	25	4810	0.83	22.3	2.29	0.07	17′ [50]
**UiO-66-NH_2_**	PIM-1	10	4	25	2869	−6.1	27.5	70.8	1.48	17′ [66]
**UiO-66-NH_2_**	PIM-1	10	4 ^(c)^	25	1900	−37.8	24	49.0	0.67	17′ [66]
**UiO-66-NH_2_**	PIM-1 (3-month aging)	9.1	1 ^(e)^	25	4835	1.36	28.2	29.4	0.75	17′ [50]
**UiO-66**	PU	24	-	-	75.2	95.8	34.2	−12.8	0.27	18′ [52]
**UiO-66**	Matrimid	10	4	37	7.8	13.0	29.4	−1.4	0.08	18′ [65]
**Azo-UiO-66**	Matrimid	10	4	37	10	44.9	37	24.0	0.99	18′ [65]
**UiO-66**	ODPA-TMPDA	20	1 ^(f)^	35	169	92.0	31.9	−3.6	0.54	This work
**UiO-66-NH_2_**	ODPA-TMPDA	20	1 ^(f)^	35	142	61.3	37.1	12.0	0.81	This work
**UiO-66-Br**	ODPA-TMPDA	20	1 ^(f)^	35	200	127	34.5	4.23	0.94	This work
**UiO-66-(OH)_2_**	ODPA-TMPDA	20	1 ^(f)^	35	125	42.0	38.9	17.5	0.81	This work

**Note:** % En.—Percentage enhancement; *F*_index_—Filler enhancement index (in Section 2.4.5); MeOH—methanol; PEBA—polyether block amide; PSF—Polysulfone; PU—Polyurethane ether; azo—azobenzene; UiO-66-ref—UiO-66 particles without water modulation (to make small particles). ^(a)^ The performance in the table is reported as pure gas permeation unless stated (indicated in the pressure column); ^(b)^ the loading amount is selected to be as close as possible to the amount that is used in this work in order to provide a better comparability with the *F*_index_ calculation; ^(c)^ CO_2_/N_2_ (50/50, in vol%); ^(d)^ humid condition; ^(e)^ N_2_/CO_2_/O_2_ (80:10:10, in vol%); ^(f)^ N_2_/CO_2_ (80:20, in vol%).

## Supplementary Materials

The following are available online at https://www.mdpi.com/2077-0375/10/7/154/s1, Figure S1: EDX analysis of UiO-66-Br, Figure S2: (**a**) CO_2_ adsorption at 25 °C; (**b**) N_2_ adsorption at 25 °C; (**c**) Isosteric heat of adsorption of CO_2_ and (**d**) CO_2_/N_2_ IAST selectivity (feed mixture of CO_2_/N_2_ = 20/80) for UiO-66, UiO-66-NH_2_, UiO-66-Br and UiO-66-(OH)_2_, Figure S3: FTIR spectrum of mixed-matrix membrane, Figure S4: XRD analysis of mixed-matrix membrane, Figure S5: TGA analysis of mixed-matrix membrane, Figure S6: Comparison of the gas permeation data with the upper bound limit for CO_2_/N_2_ constructed in 2008. The numerical number indicated in the figure illustrate the value of *F_index_* as described in Section 2.4.5. The data used in this plot is provided in Table 4, Table S1: Elemental analysis of UiO-66 and its derivative, Table S2: Theoretical amount of UiO-66 and its derivative, Table S3: Fitting parameters for CO_2_ and N_2_ for UiO-66 and its derivative at 25 °C, Table S4: Fitting parameters for CO_2_ and N_2_ for UiO-66 and its derivative at 35 °C, Table S5: Fitting parameters for CO_2_ and N_2_ for polymeric and mixed-matrix membranes at 35 °C.
