# Supplementary material for: CO2/N2 Separation Properties of Polyimide-Based Mixed-Matrix Membranes Comprising UiO-66 with Various Functionalities
Source: Membranes (Basel). 2020 Jul 17;10(7):154. doi: 10.3390/membranes10070154 (PMC7407110; doi:10.3390/membranes10070154)
Supplement: Supplementary file 1 [file membranes-10-00154-s001.pdf]

Supplementary Information

for

CO_2/N_2 separation properties of polyimide-based composite membranes comprising UiO-66 with various functionalities

Chong Yang Chuah[1], Junghyun Lee[2], Juha Song[2], and Tae-Hyun Bae[3,*]

[1] Singapore Membrane Technology Centre, Nanyang Environment and Water Research Institute, Nanyang Technological University, Singapore 637141, Singapore

[2] School of Chemical and Biomedical Engineering, Nanyang Technological University, Singapore 637459, Singapore

[3] Department of Chemical and Biomolecular Engineering, Korea Advanced Institute of Science and Technology, Daejeon 31414, Republic of Korea

* Corresponding author

E-mail: thbae@kaist.ac.kr

Table S1 Elemental Analysis of UiO-66 and its derivative

Sample	C (%)	H (%)	N (%)	Br (%)[a]
**UiO-66**	29.75	2.121	0.899	-
**UiO-66-NH_2**	28.76	2.833	3.845	-
**UiO-66-Br**	22.93	1.772	0.259	9.262
**UiO-66-$(OH)_2$**	28.77	1.811	1.834	-

[a] Estimated from EDX analysis (Figure S1)

Table S2 Theoretical amount of UiO-66 and its derivative(a)

Sample(b)	M_w (g/mol)	Zr (%)	C (%)	H (%)	O (%)	N (%)	Br (%)
**UiO-66**	1664.1	32.89	34.64	1.695	30.77	0	0
**UiO-66-NH_2**	1712.1	31.96	33.67	1.413	28.03	4.91	0
**UiO-66-Br**	2137.4	25.61	26.97	1.037	23.95	0	22.43
**UiO-66-$(OH)_2$**	1856.0	29.48	31.06	1.520	37.93	0	0

(a) Due to the potential presence of residual solvents or water in MOF, it is possible that the deviation between experimental and theoretical value can be observed. Nevertheless, the trend in C(%), H(%), N(%) are generally consistent across all UiO-66 framework.

(b) Molecular formula: $Zr_6C_{48}H_{28}O_{32}$ (UiO-66); $Zr_6C_{48}H_{24}O_{30}N_6$ (UiO-66-NH_2); $Zr_6C_{48}H_{22}O_{32}Br_6$ (UiO-66-Br); $Zr_6C_{48}H_{28}O_{44}$ (UiO-66-$(OH)_2$

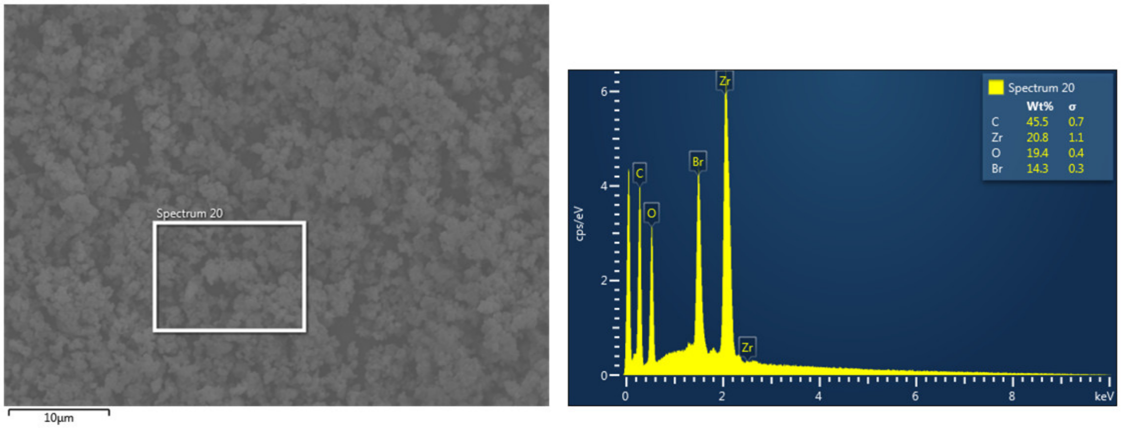

Figure S1 EDX analysis of UiO-66-Br

Table S3 Fitting Parameters for CO_2 and N_2 for UiO-66 and its derivative at 25 ^{o}C

Unit of P = bar; Unit of q = mmol/g

$$q = \frac{q_{sat} bp}{1 + bp}$$

Sample	*gas*	q_{sat} *(bar)*	*b* *(bar^{-1})*	R^2 value
**UiO-66**	CO_2	6.119	0.2734	1
	N_2	6.119	0.01543	0.992
**UiO-66-NH_2**	CO_2	5.376	0.359	0.9998
	N_2	5.376	0.01673	0.964
**UiO-66-Br**	CO_2	3.302	0.837	0.9998
	N_2	3.302	0.03773	0.9972
**UiO-66-$(OH)_2$**	CO_2	2.151	1.040	0.9996
	N_2	2.151	0.0271	0.9889

Table S4 Fitting Parameters for CO_2 and N_2 for UiO-66 and its derivative at 35 ^{o}C

Unit of P = bar; Unit of q = mmol/g

$$q = \frac{q_{sat} bp}{1 + bp}$$

Sample	*gas*	q_{sat} *(bar)*	*b (bar^{-1})*	R^2 value
**UiO-66**	CO_2	5.404	0.2357	0.9998
	N_2	5.404	0.01432	0.9894
**UiO-66-NH_2**	CO_2	5.528	0.2526	0.9996
	N_2	5.528	0.01228	0.9818
**UiO-66-Br**	CO_2	3.405	0.5721	0.9995
	N_2	3.405	0.02908	0.9947
**UiO-66-$(OH)_2$**	CO_2	2.065	0.7765	0.9994
	N_2	2.065	0.02145	0.9800

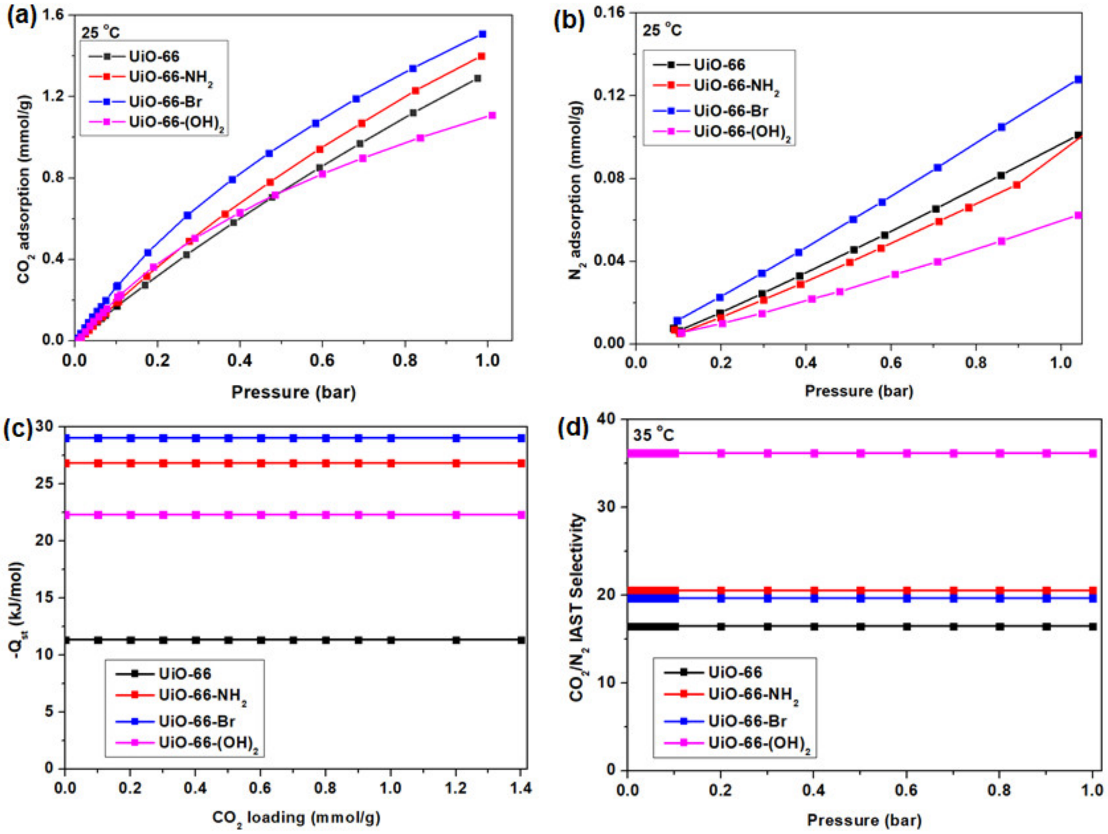

Figure S2 (a) CO_2 adsorption at 25 °C; **(b)** N_2 adsorption at 25 °C; **(c)** Isosteric heat of adsorption of CO_2 and **(d)** CO_2/N_2 IAST selectivity (feed mixture of CO_2/N_2 = 20/80) of for UiO-66, UiO-66-NH_2, UiO-66-Br and UiO-66-$(OH)_2$

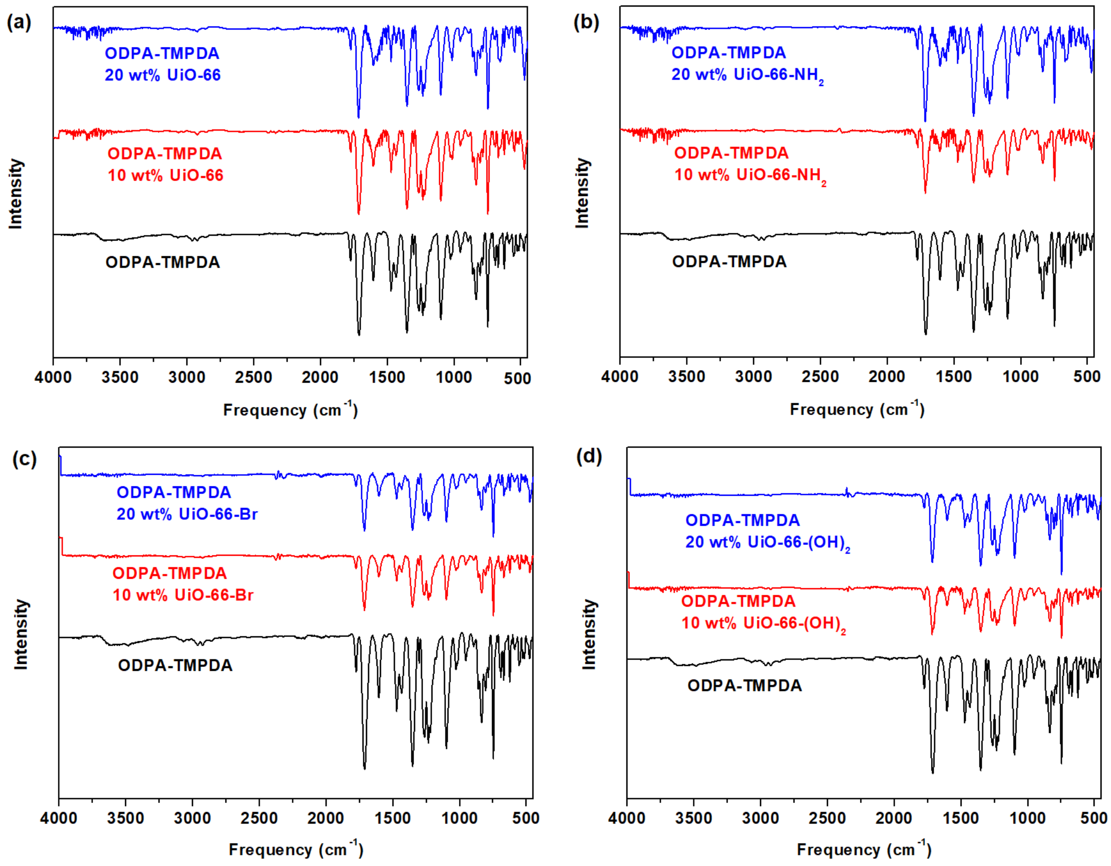

Figure S3 FT-IR spectrum of mixed-matrix membrane

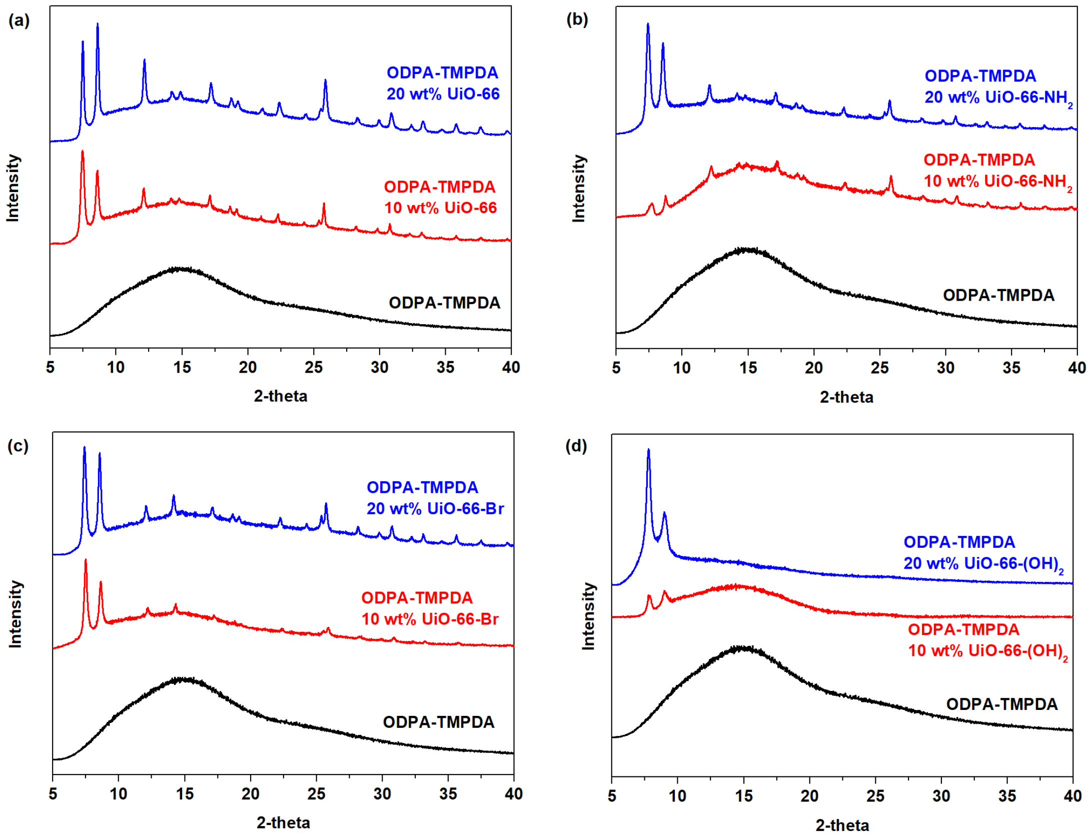

Figure S4 XRD analysis of mixed-matrix membrane

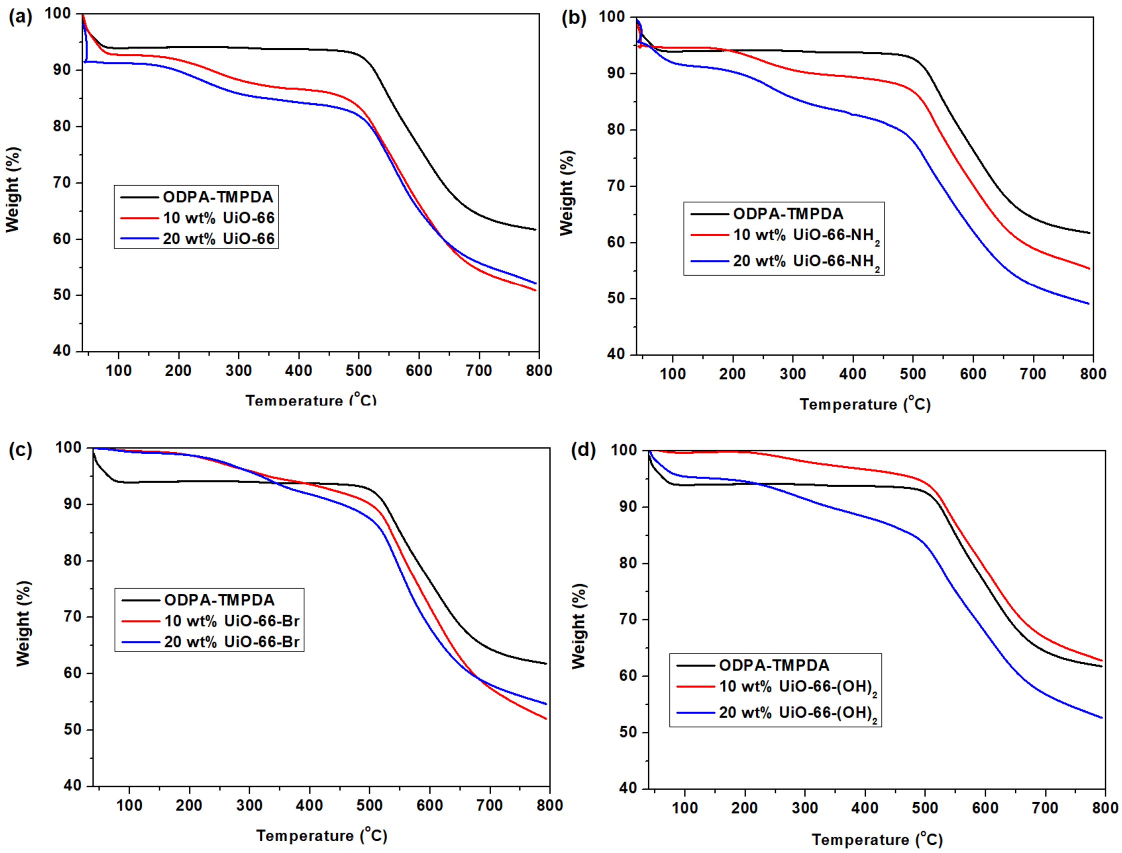

Figure S5 TGA analysis of mixed-matrix membrane

Table S5 Fitting Parameters for CO_2 and N_2 for polymeric and mixed-matrix membranes at 35 ^oC

Unit of P = bar; Unit of q = mmol/g

$$q = \frac{q_{sat} bp}{1 + bp}$$

Sample	*gas*	q_{sat} *(bar)*	*b (bar⁻¹)*	R^2 value
**ODPA-TMPDA**	CO_2	1.444	0.9832	0.9996
	N_2	1.017	0.02502	0.9862
**20 wt% UiO-66**	CO_2	1.611	0.6785	0.9999
	N_2	0.5807	0.04649	0.9778
**20 wt% UiO-66-NH_2**	CO_2	1.446	0.6945	0.9999
	N_2	0.1717	0.1365	0.9869
**20 wt% UiO-66-Br**	CO_2	1.381	0.7766	0.9999
	N_2	1.471	0.01143	0.9451
**20 wt% UiO-66-$(OH)_2$**	CO_2	1.373	0.8565	0.9998
	N_2	4.263	0.005002	0.9847

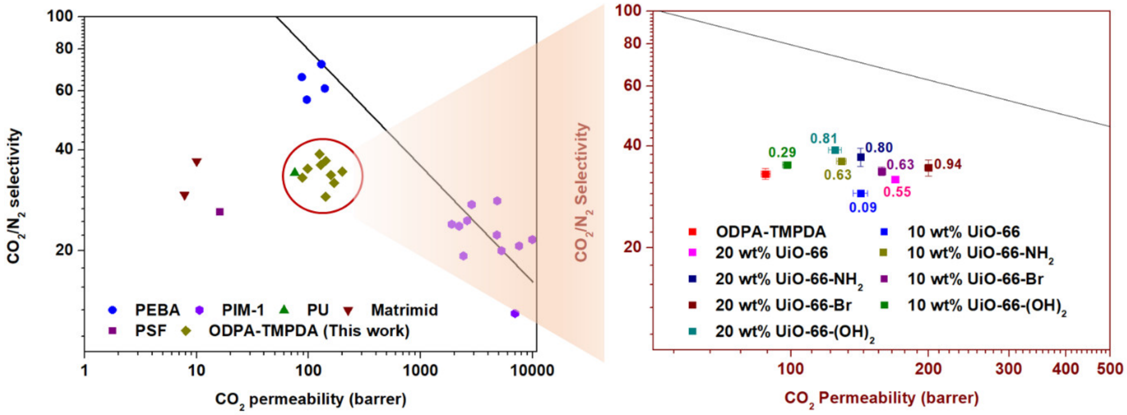

Figure S6 Comparison of the gas permeation data with the upper bound limit for CO_2/N_2 constructed in 2008. The numerical number indicated in the figure illustrate the value of F_{index} as described in Section 2.4.5. The data used in this plot is provided in **Table 4**.
